# Cerebral white matter hyperintensities indicate severity and progression of coronary artery calcification

**DOI:** 10.1038/s41598-024-55305-0

**Published:** 2024-02-26

**Authors:** Markus Kneihsl, Thomas Gattringer, Edith Hofer, Peter P. Rainer, Gerhard Ranner, Simon Fandler-Höfler, Melanie Haidegger, Sabine Perl, Christian Enzinger, Reinhold Schmidt

**Affiliations:** 1 Department of Neurology, Medical University of Graz, Auenbruggerplatz 22, 8036 Graz, Austria; 2 Division of Neuroradiology, Vascular and Interventional Radiology, Department of Radiology, Medical University of Graz, Graz, Austria; 3 Institute for Medical Informatics, Statistics and Documentation, Medical University of Graz, Graz, Austria; 4 Division of Cardiology, Department of Internal Medicine, Medical University of Graz, Graz, Austria; 5 BioTechMed Graz, Graz, Austria; 6 CT and MR Imaging Center Geidorf, Graz, Austria

**Keywords:** Coronary artery calcification, White matter hyperintensities, Cerebral small vessel disease, Aging, Neuro-vascular interactions, Cardiovascular diseases

## Abstract

Cerebral white matter hyperintensities (WMH) have been associated with subclinical atherosclerosis including coronary artery calcification (CAC). However, previous studies on this association are limited by only cross-sectional analysis. We aimed to explore the relationship between WMH and CAC in elderly individuals both cross-sectionally and longitudinally. The study population consisted of elderly stroke- and dementia-free participants from the community-based Austrian Stroke Prevention Family Study (ASPFS). WMH volume and CAC levels (via Agatston score) were analyzed at baseline and after a 6-year follow-up period. Of 324 study participants (median age: 68 years), 115 underwent follow-up. Baseline WMH volume (median: 4.1 cm^3^) positively correlated with baseline CAC levels in multivariable analysis correcting for common vascular risk factors (p = 0.010). While baseline CAC levels were not predictive for WMH progression (p = 0.447), baseline WMH volume was associated CAC progression (median Agatston score progression: 27) in multivariable analysis (ß = 66.3 ± 22.3 [per cm^3^], p = 0.004). Ten of 11 participants (91%) with severe WMH (Fazekas Scale: 3) at baseline showed significant CAC progression > 100 during follow-up. In this community-based cohort of elderly individuals, WMH were associated with CAC and predictive of its progression over a 6-year follow-up. Screening for coronary artery disease might be considered in people with more severe WMH.

## Introduction

Cerebral white matter hyperintensities (WMH) of presumed vascular origin are considered as a hallmark neuroimaging feature of cerebral small vessel disease and a frequent incidental finding on brain MRI^1,2^. WMH are well-known to increase the risk for stroke, dementia and permanent disability, and are associated with long-term mortality^3^. Chronic hypoperfusion and ischemia in white matter regions, attributed to alterations in cerebral arterioles and capillaries, have been identified as key components of small vessel disease-related WMH^4–7^

Moreover, WMH have also been associated with subclinical atherosclerotic large vessel disease^8,9^. In particular coronary artery calcification (CAC), a well-known marker of coronary artery disease, has been related to WMH severity^8–11^. Apart from age, genetic predisposition, and classical vascular risk factors, previous studies suggested a more direct pathophysiological link between atherosclerotic large vessel disease and cerebral WMH^9,12^. Vessel wall stiffening of brain-supplying arteries was suspected to induce a more pulsatile blood flow that causes cerebral tissue damage and promotes WMH^9,12,13^. Consequently, coronary artery atherosclerosis has been postulated as a potential early indicator of WMH progression^9^. However, all studies published thus far are limited by their cross-sectional design^8–11^.

In this study, our objective was to investigate the association between WMH and coronary atherosclerotic disease, as indicated by CAC, over a long-term follow-up period in a stroke- and dementia-free elderly population.

## Results

**Figure 1:**
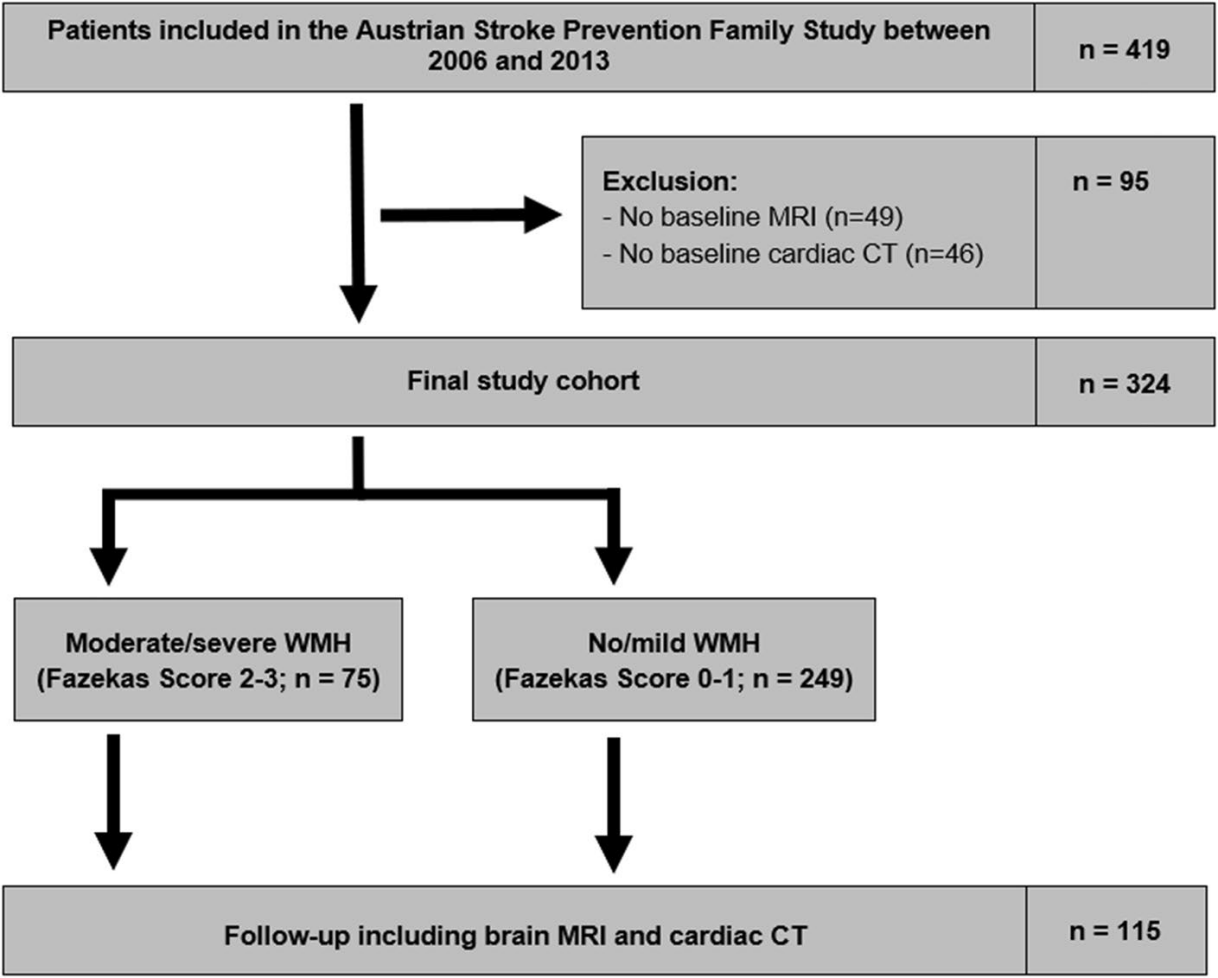
Flow diagram of included study participants.

A total of 419 participants was included in the ASPFS in the studied period. Of those, 95 participants had to be excluded due to the unavailability of cardiac CT or brain MRI at baseline (22.7%). This resulted in a final study cohort of 324 participants (median age: 68 years; female: 59.8%) (Fig. 1).

### Baseline

Of all included study participants, 75 (23.1%) had moderate/severe WMH on brain MRI at baseline. Median WMH volume was 4.1 cm^3^ (median PVWMH: 2.9 cm^3^; median DWMH: 0.9 cm^3^). Median baseline CAC level measured by the Agatston score was 7 (interquartile range, IQR: 112) and 37 participants fulfilled the criteria for severe calcification of the coronary arteries (Agatston score > 400, 8.8%).

In univariable analysis, WMH volume correlated with age (r_s_: 0.421, p < 0.001), arterial hypertension (r_s_: 0.279, p < 0.001), GFR (r_s_: − 0.189, p < 0.001), lacunes (r_s_: 0.362, p < 0.001), old cortical infarcts (r_s_: 0.204, p < 0.001) on baseline MRI, and baseline Agatston score (r_s_: 0.253, p < 0.001) (Table 1).

**Table 1:** Demographic and clinical data of ASPFS participants according to qualitative and quantitative WMH burden at baseline.

Variable/clinical finding	ASPFS total cohort (n = 324)	Moderate/severe WMH (Fazekas 2–3) (n = 75)	No/mild WMH (Fazekas 0–1) (n = 249)	p-value^1^	WMH volume at baseline (Spearman rank correlation, r_s_)	p-value^1^
Demographics						
Age, years (median, IQR)	68 (16)	71 (9)	66 (13)	< 0.001	0.421	< 0.001
Female sex, n. %	198 (59.8)	53 (67.9)	145 (57.3)	0.094	0.071	0.198
Medical history, n. %						
Arterial hypertension	213 (64.4)	62 (79.5)	151 (59.7)	0.001	0.279	< 0.001
Dyslipidemia	251 (75.8)	59 (75.6)	192 (75.9)	0.964	0.036	0.518
Diabetes mellitus	33 (10.0)	12 (15.4)	21 (8.3)	0.068	0.115	0.037
Smoking (active or former)	74 (22.8)	18 (23.1)	57 (22.5)	0.920	− 0.064	0.248
Myocardial infarction	12 (3.6)	5 (6.4)	7 (2.8)	0.132	0.011	0.844
Atrial fibrillation	16 (4.8)	6 (7.7)	10 (4.0)	0.178	0.097	0.078
Left ventricular hypertrophy	27 (8.2)	6 (7.7)	21 (8.3)	0.864	0.078	0.401
Glomerular filtration rate (median, IQR)	72.0 (11.1)	69.2 (14.6)	72.9 (17.2)	0.032	− 0.189	< 0.001
MRI parameters, n. %						
WMH volume (cm^3^, median, IQR)	4.1 (5.9)	15.6 (13.4)	3.0 (3.0)	< 0.001	NA	NA
PVH WMH volume (cm^3^, median, IQR)	2.9 (3.0)	9.1 (7.7)	2.1 (7.9)	< 0.001	NA	NA
Deep WMH volume (cm^3^, median, IQR)	0.9 (3.2)	9.4 (8.7)	0.6 (1.5)	< 0.001	NA	NA
≥ 1 lacune	33 (10.0)	24 (30.8)	11 (4.3)	< 0.001	0.362	< 0.001
Old cortical infarct	16 (4.8)	11 (14.1)	5 (2.0)	< 0.001	0.204	< 0.001
Coronary artery disease						
Agatston score (mean, SD)	7 (112)	44 (178)	9 (135)	< 0.001	0.253	< 0.001
Follow-up^a^, n. %						
Median follow-up period, years	5.8 (0.7)	5.8 (0.6)	5.8 (0.8)	0.904	0.036	0.781
Vascular event during follow-up (TIA/stroke/MCI)	14 (4.2)	6 (7.7)	8 (3.2)	0.082	0.148	0.007
Agatston score progression during follow-up (median, IQR)	27 (177)	93 (325)	23 (150)	0.043	0.383	< 0.001

MRI: magnetic resonance imaging; ASPFS: Austrian stroke prevention family study; WMH: White matter hyperintensities; SD: standard deviation; TIA: transient ischemic attack; MCI: myocardial infarction.

^1^Demonstrated p-value was determined by comparing participants with moderate/severe WMH to those with no/mild WMH.

^a^Follow-up was available in 115 participants.

In multivariable linear regression analysis, age (ß: 0.19 ± 0.08 [per year], p = 0.011), arterial hypertension (ß: 3.89 ± 1.56, p = 0.015) and baseline Agatston score (ß: 0.60 ± 2.01 [per 100 points], p = 0.010) remained positively associated with baseline WMH volume after adjusting for important co-variables (Table 2). When employing the same model with PVWMH or DWMH volume as the target variable, instead of the total WMH volume, only PVWMH volume exhibited a significant association with the baseline Agatston score (ß: 0.40 ± 0.10, p = 0.008; DMWH, ß: 0.30 ± 0.10, p = 0.079).

**Table 2:** Multivariable linear regression analysis with baseline WMH load (in cm^3^) as the target variable.

	ß (SE)	95% confidence interval	p-value
Age (per year)	0.19 (0.08)	0.0 to 0.3	0.011
Female sex	2.39 (1.54)	− 0.6 to 5.4	0.121
Arterial hypertension	3.89 (1.56)	0.8 to 7.0	0.015
Diabetes mellitus	0.59 (2.47)	− 4.3 to 5.5	0.809
Agatston score at baseline (per 100 points)	0.60 (2.01)	0.2 to 1.1	0.010

WMH: white matter hyperintensities; ASPFS: Austrian stroke prevention family study; SE: standard error; MCA: MRI: magnetic resonance imaging; MCA: middle cerebral artery.

### Follow-up

115 participants underwent a median long-term follow-up of 5.8 years (range: 5.2–6.4 years) including repeated brain MRI and CT of the coronary arteries. Vascular events occurred in 14 individuals during the follow-up period (12.1%; myocardial infarction: n = 7, stroke: n = 2, TIA: n = 5).

Median progression of the Agatston score during follow-up was 27 (IQR: 177) and median WMH volume progression from baseline to follow-up was 0.4 cm^3^ (IQR: 0.7; WMH progression of ≥ 1 score points on the Fazekas Scale: n = 24, 20.9%). CAC progression was associated with age (r_s_ = 0.281, p < 0.001), arterial hypertension at baseline (r_s_ = 0.354, p < 0.001), diabetes mellitus (r_s_ = 0.187, p = 0.045), decreased GFR (r_s_ = − 0.235, p = 0.012) as well as baseline WMH volume (r_s_ = 0.383, p < 0.001) and baseline Agatston score (r_s_ = 0.898, p < 0.001) (Table 3). Arterial hyertension as well as increased blood glucose and cholesterol levels during the follow-up period were not associated with CAC progression (p > 0.05 each).

**Table 3:** Demographic and clinical data of ASPFS participants according to CAC progression.

Variable/clinical finding	CAC progression (upper quartile, n = 28)	CAC progression (quartiles 1–3, n = 87)	p-value^1^	CAC progression during follow-up (Spearman rank correlation, r_s_)	p-value^1^
Demographics					
Age, years (median, IQR)	70 (6)	66 (14)	< 0.001	0.281	< 0.001
Female sex, n. %	10 (35.7)	47 (54.0)	0.092	− 0.133	0.158
Medical history, n. %					
Arterial hypertension	26 (92.9)	50 (57.5)	< 0.001	0.354	< 0.001
Dyslipidemia	22 (78.6)	64 (73.6)	0.596	0.154	0.100
Diabetes mellitus	4 (14.3)	8 (9.2)	0.443	0.187	0.045
Smoking (active or former)	8 (28.6)	20 (23.0)	0.549	0.116	0.218
Glomerular filtration rate (median, IQR)	68.7 (12.5)	74.7 (17.9)	0.036	− 0.235	0.012
MRI parameters at baseline, n. %					
WMH volume (cm^3^, median, IQR)	6.3 (11.7)	3.8 (4.5)	0.012	0.383	< 0.001
PVH WMH volume (cm^3^, median, IQR)	3.6 (4.0)	2.7 (2.9)	0.028	0.233	0.012
Deep WMH volume (cm^3^, median, IQR)	1.7 (9.6)	0.8 (2.9)	0.015	0.400	< 0.001
WMH (median, IQR)	1 (2)	1 (2)	0.003	0.280	0.002
Moderate/severe WMH (Fazekas 2–3)	9 (32.1)	15 (17.2)	0.091	0.161	0.065
Severe WMH (Fazekas 3)	8 (29.6)	3 (3.6)	< 0.001	0.330	< 0.001
≥ 1 lacune	9 (32.1)	6 (6.9)	< 0.001	0.362	< 0.001
Old cortical infarct	2 (7.1)	0 (0.0)	0.012	0.204	< 0.001
Coronary artery disease					
Agatston score at baseline (mean, IQR)	273 (537)	3 (44)	< 0.001	0.898	< 0.001
Follow-up^a^, n. %					
Follow-up period, years (median, IQR)	5.8 ± 0.7	5.8 ± 0.7	0.755	0.046	0.822
Arterial hypertension during FU	16 (57.1)	32 (37.2)	0.064	0.174	0.059
LDL cholesterol > 116 mg/dl during FU	9 (32.1)	38 (44.2)	0.261	− 0.105	0.265
Fasting blood glucose level > 126 mg/dl during FU	2 (7.1)	2 (2.3)	0.229	0.113	0.233
Smoking during FU	3 (11.1)	13 (15.1)	0.603	− 0.049	0.606
WMH progression during FU (quantitative: cm^3^, median, IQR)	0.4 (0.7)	0.5 (0.9)	0.475	− 0.058	0.562
WMH progression during FU (qualitative: ≥ 1 category after Fazekas)	4 (19.1)	20 (22.2)	0.322	− 0.133	0.186
Vascular event during FU (TIA/stroke/MCI)	4 (14.3)	10 (11.5)	0.694	0.031	0.745

MRI: magnetic resonance imaging; ASPFS: Austrian stroke prevention family study; WMH: white matter hyperintensities; SD: standard deviation; TIA: transient ischemic attack; MCI: myocardial infarction; LDL: low density lipoprotein; FU: follow-up.

^1^Demonstrated p-value was determined by comparing participants with moderate/severe WMH to those with no/mild WMH.

^a^Follow-up was available in 115 participants.

In multivariable linear regression analysis, WMH volume at baseline remained significantly associated with CAC progression (ß = 66.3 ± 22.3 [per cm^3^], p = 0.004) as the sole variable, in addition to baseline Agatston score (ß = 50.2. ± 6.2 [per 100 points], p < 0.001) (Table 4). Again, the model was run with DWMH and PVWMH instead of total WMH volume: Both WMH subtypes tended to be predictive for CAC progression (DWMH, ß:12.0 ± 7.0, p = 0.097; PVWMH, ß:27.4 ± 16.2, p = 0.096). Of note, 10 of 11 participants with severe baseline WMH according to a Fazekas scale score of 3 had CAC progression of > 100 over the follow-up period (90.9% vs. 28.3%, p < 0.001).

**Table 4:** Predictors of CAC progression in multivariable linear regression analysis.

	ß (SE)	95% confidence interval	p-value
Age (per year)	− 0.04 (0.08)	− 0.2 to 0.2	0.590
Female sex	3.31 (4.18)	− 4.9 to 11.6	0.429
Arterial hypertension	4.12 (4.61)	− 5.1 to 13.3	0.377
Diabetes mellitus	3.26 (6.52)	− 9.7 to 16.2	0.621
Agatston score at baseline (per 100 points)	50.20 (6.21)	41.80 to 67.94	< 0.001
WMH volume at baseline (per cm^3^)	66.30 (22.31)	22.41 to 111.00	0.004
PVWMH volume at baseline (per cm^3^)^a^	27.41 (16.24)	− 2.73 to 66.58	0.097
DWMH volume at baseline (per cm^3^)^a^	12.02 (7.01)	− 1.10 to 26.21	0.096

WMH: white matter hyperintensities; PVWMH: periventricular white matter hyperintensities, DWMH: deep white matter hyperintensities; SE: standard error; MCA: MRI: magnetic resonance imaging.

^a^Multivariable linear regression model was recalculated with both WMH subtype volumes (PVWMH, DWMH) instead of total WMH volume.

**Figure 2:**
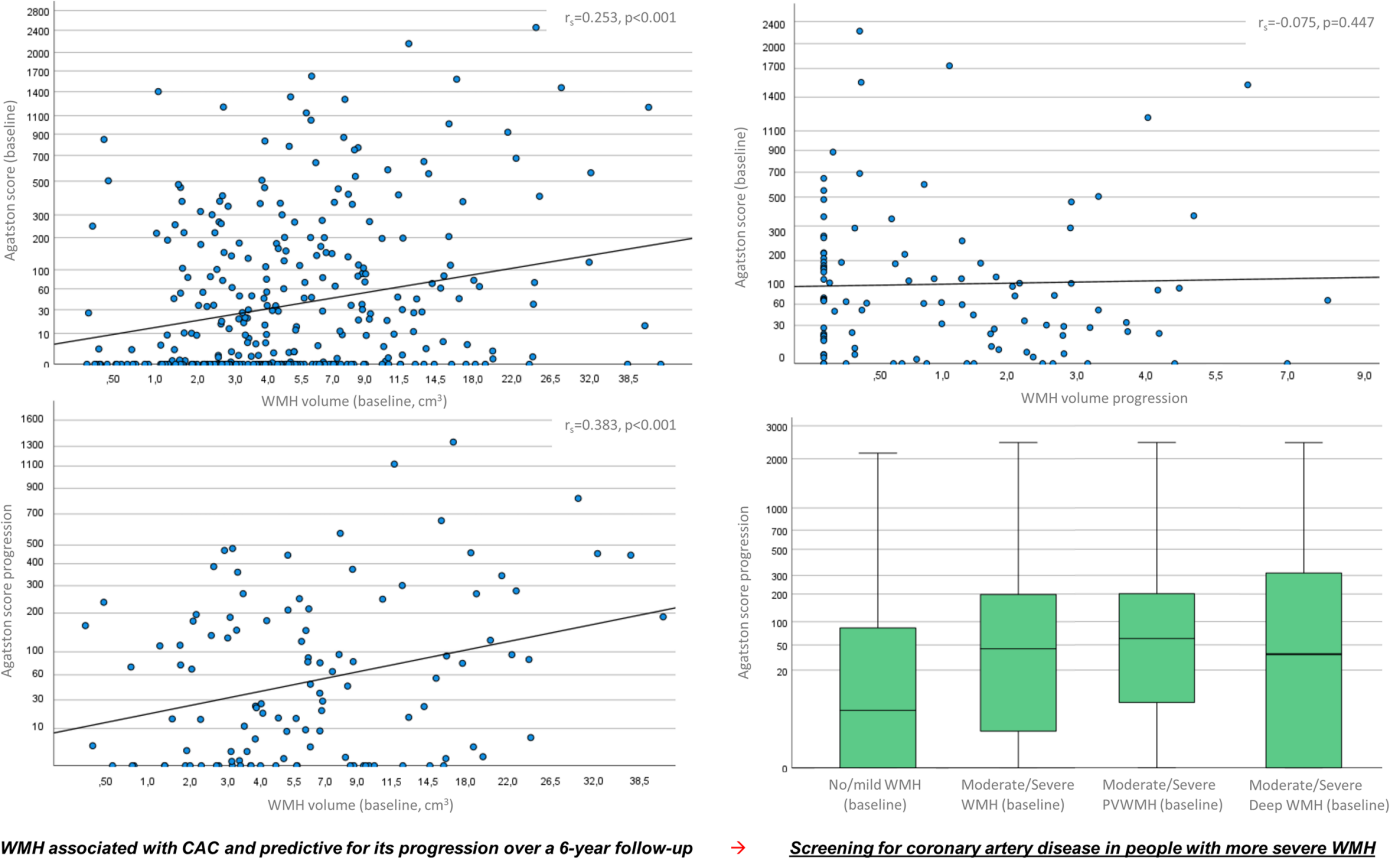
Correlations between Agatston score and WMH volume at baseline and their progression over a long-term follow-up period of 6 years.

Vice versa, there was no association between CAC at baseline and WMH progression during the follow-period (r_s_ = − 0.075, p = 0.447, univariable analysis) and even high baseline CAC scores > 400 (n = 29) did not predict a significant WMH progression during the follow-up period (WMH progression: 1.19 ± 1.66 vs. 1.07 ± 2.62, p = 0.884) (Fig. 2). Similarly, no associations were found between baseline CAC scores and WMH progression in both WMH subtypes (PVWMH progression: r_s_ = − 0.092, p = 0.672; DWMH progression: r_s_ = − 0.022, p = 0.801).

## Discussion

In this cohort of stroke- and dementia-free elderly people, WMH volume on brain MRI was associated with calcification of the coronary arteries at baseline and predictive for its progression over long-term follow-up. Individuals with severe WMH, as indicated by a Fazekas score of 3, are at high risk for a substantial progression of CAC.

Our results are of interest as previous studies led to speculations about the mechanisms behind the association of cerebral WMH—a hallmark feature of cerebral small vessel disease—and (subclinical) large vessel atherosclerosis such as CAC^8–11^. A combination of shared classical vascular risk factors (predominantly arterial hypertension) and a genetic predisposition was suspected behind this phenomenon^8–11^. In this context, Johansen et al. identified differences in the strengths of the relationship between CAC and different WMH subtypes^9^. The more pronounced association observed between CAC and PVWMH, as opposed to DWMH, might be attributed to the typical vascular architecture of PVWMH-related short penetrating microvessels. These vessels may be more directly affected by arterial hypertension compared to the longer microvessels that supply the deep white matter^14,15^. Atherosclerotic changes in the penetrating branches of the large intracranial arteries might lead to hypoperfusion and ischemia, representing a key mechanism underlying WMH development^16^. In addition, similar genetic variations were identified in patients with calcification of coronary arteries and a high burden of PVWMH^17^.

In our analysis, PVWMH also showed a stronger correlation with baseline CAC levels (in multivariable analysis), but the difference between both WMH subtypes was rather small in absolute numbers. Future studies might analyze ultrastructural white matter changes using diffusion tensor imaging (DTI)^18^ and their association with macrovascular disease to improve the pathophysiological understanding of the presented association.

The unique longitudinal design of this study further allowed us to investigate potential associations between CAC as marker of (subclinical) atherosclerotic large vessel disease and WMH over a long-term follow-up period of 6 years. Apart from genetics and shared vascular risk factors, previous studies indicated a more direct link explaining the association between CAC and WMH. As atherosclerotic large vessel disease results in the stiffening of arteries, it is hypothesized that the subsequent increase in blood-flow pulsatility within the brain-supplying vessels directly damages the small cerebral vasculature, thereby promoting WMH^9,12,13^. For this reason, CAC was assumed to predict cerebral WMH progression, but previous studies only had cross-sectional data available^8–11^.

Our results do not support this hypothesis as we did not identify an association between CAC levels at baseline and WMH progression (neither total WMH nor WMH subtypes) during the follow-up period. Moreover, even participants with severe calcification of the coronary arteries at baseline (CAC score > 400) did not show a more pronounced WMH progression.

These results align with recently published data that failed to establish an association between intracranial pulsatility, measured in the middle cerebral artery, and both WMH volume at baseline and WMH progression during follow-up^19^.

Most notably, we found that baseline WMH volume predicted CAC progression during the follow-up period. Specifically, ten out of eleven participants with severe baseline WMH, as indicated by a Fazekas score of 3, showed substantial CAC progression of > 100. PVWMH and DWMH had a similar predictive value for CAC progression. Apart from a shared genetic predisposition, classical vascular risk factors (i.e., arterial hypertension) might be important factors behind this relation. In this context, all participants with severe baseline WMH (Fazekas 3) and CAC progression during follow-up also had underlying arterial hypertension. Our results therefore point towards a subgroup of WMH patients, in which intense and continuous vascular risk factor control might be crucial to avoid further damage of the brain, but also to avoid macrovascular changes in other vascular beds such as the coronary arteries. Moreover, treating physicians should be aware of signs and symptoms of cardiac disease in patients with high WMH load and initiate cardiological exploration or even coronary artery disease screening in case of any clinical suspicion.

## Limitations

Our study is limited by the fact that only a subgroup of study participants (27%) underwent follow-up brain MRI and coronary artery CT (n = 115). However, this should not have influenced our results to a relevant extent as there were no differences in demographics, vascular risk factors and CAC or WMH volume at baseline between patients with and those without follow-up (p > 0.1, data not shown). Based on the small-sized follow-up cohort, we cannot exclude that we have overlooked a (small) predictive value of baseline CAC levels on WMH progression. We also only observed few outcome events in this study not allowing to report on the predictive value of WMH, CAC and associated vascular events, which should be addressed in larger prospective studies.

## Conclusions

This study reinforces the correlation between cerebral WMH and large artery atherosclerosis. Moreover, WMH serve as predictors for the progression of coronary artery disease during a long-term follow-up period. Clinicians should be aware of this observed association and may consider to screen individuals with severe WMH for coronary artery disease. Intense control of vascular risk factors is essential for all such patients.

## Materials and methods

All methods were performed in accordance with the relevant guidelines and regulations.

The study was approved by the ethics committee of the Medical University of Graz (Approval number: 17-088 ex 05/06). Written informed consent was obtained by all included study participants.

### Selection of participants and data collection

All included study participants derive from the Austrian Stroke Prevention Family Study (ASPFS), an extension of the Austrian Stroke Prevention Study (ASPS). ASPFS is a prospective population-based study that was designed to assess the effects of vascular risk factors on brain structure, function and vessel damage in different vascular beds^20^. Between 2006 and 2013, study participants of the original study—ASPS—and their first-degree relatives were invited to enter the ASPFS study. Inclusion criteria included the absence of a history of cerebrovascular disease (stroke or transient ischemic attack) or dementia, as well as a normal neurological examination.

This is a single-center study. All participants were recruited at the University Hospital of Graz, Austria.

Baseline and follow-up assessments comprised blood pressure measurements, laboratory tests of vascular risk factors (such as blood glucose and serum lipid levels), a clinical evaluation of comorbidities as well as coronary artery computed tomography (CT) and brain magnetic resonance imaging (MRI). Vascular risk factors at baseline were defined according to latest guideline recommendations based on documented parameters (hypertension: systolic blood pressure > 140 mmHg, diastolic blood pressure > 90 mmHg; diabetes: fasting plasma glucose > 126 mg/dl; dyslipidemia: LDL cholesterol ≥ 116 mg/l) or if patients were already treated with respective medication (such as antihypertensives or statins)^21–23^. The guideline-based initiation of dedicated treatments was always verified by the study team. During the follow-up period, the same thresholds for increased blood pressure, hyperglycemia and dyslipidemia were used to identify patients with poor vascular risk factor control^21–23^.

### Brain imaging

All brain MRI investigations were performed on a 3.0 T scanner (TimTrio; Siemens Healthcare, Erlangen, Germany) including standard T1- and T2-weighted pulse sequences (slice thickness = 3 mm), Fluid Attenuated Inversion Recovery (FLAIR; TR = 10,000 ms, TE = 69 ms, inversion time = 2500 ms) and high resolution T1 weighted rapid acquisition gradient-echo (MPRAGE; TR = 1900 ms, TE = 2.19 ms, inversion time = 900 ms).

All MRI scans were reviewed by blinded neuroradiological experts (C.E., R.S.). WMH, lacunes and (chronic) cortical infarcts were assessed on T2 and FLAIR images. WMH severity was rated according to the Fazekas rating scale in deep and periventricular locations^24^. For quantitative assessment, WMH areas were first segmented manually and consecutively added to a total lesion volume using FMRIB Software Library (FMRIB, Oxford, UK; freely available at https://fsl.fmrib.ox.ac.uk)^25^.

### Coronary artery calcification

Participants underwent cardiac CT with a 64-channel multidetector computed tomography (GE Imatron, San Francisco, USA) at baseline and follow-up. ECG triggering was used at 80% of the cardiac cycle to obtain images (slice thickness: 3 mm; image acquisition time: 100 ms). CAC was defined as a minimum of three contiguous pixels with a CT density ≥ 130 Hounsfield units. An experienced radiologist (GR) specialized in cardiac CT imaging and blinded to clinical data read all the CT images and calculated CAC scores according to the Agatston method^26^.

### Patient and public involvement

Patients or the public were not involved in the design, conduct, reporting or dissemination plans of our research.

### Statistics

Statistical analyses were performed using IBM SPSS Statistics, version 28. Aside from analyzing WMH volumes and CAC levels as continuous variables, WMH were dichotomized using the Fazekas visual grading scale scores 0–1 (i.e., no or mild WMH) versus 2–3 (i.e., moderate to severe WMH))^27^. If WMH in either deep (DWMH) or periventricular (PVWMH) location were graded as Fazekas 2 or higher, they were classified as moderate to severe WMH. In a second step, participants were divided according to CAC score severity (quartile 4 versus quartiles 1–3). Pearson’s chi-square or Fisher’s exact test was used to compare dichotomous variables. All quantitative variables were first tested for Gaussian distribution with the Kolmogorov–Smirnov test and, if Gaussian distribution was identified, a two-sample independent t-test was utilized to compare the variables. The Mann–Whitney-U-Test was used for non-parametric data.

As WMH volumes and CAC levels at baseline and follow-up were not normally distributed, Spearman’s rank correlation was performed for bivariable correlations including these parameters. A p-value less than 0.05 was considered statistically significant.

A multivariable linear regression model was fitted to identify factors that were independently associated with WMH volume and CAC levels at baseline.

Besides age and sex, the model included variables that were related to baseline WMH (target variable) and baseline CAC volume in univariable analysis (p < 0.05): arterial hypertension, diabetes and glomerular filtration rate (GFR). However, after testing for multicollinearity and interactions, GFR was removed from the multivariable analysis because of its strong correlation with age (variance inflation index > 10).

In a second step, the same model was again calculated with DWMH and PVWMH as the target variables (instead of total WMH volume) to test for the influence of different WMH subtypes.

For follow-up analyses, predictors for WMH or CAC progression were first analyzed via univariable statistics as described above. Based on these analyses, CAC progression was set as the target variable in a multivariable linear regression analysis. The model included age and sex, and further variables that were predictive for CAC progression in previous studies or in univariable analysis (arterial hypertension, diabetes, GFR, baseline WMH volume)^8–11^. Again, GFR had to be excluded based on a variance inflation index > 10 and the model was also recalculated twice including baseline volume of DWMH and PVWMH instead of (total) baseline WMH volume.

## Data Availability

Study data that support the findings of this study are available from the corresponding author upon reasonable request.
